# Standardization of an in vitro assay matrix to assess cytotoxicity of organic nanocarriers: a pilot interlaboratory comparison

**DOI:** 10.1007/s13346-022-01203-9

**Published:** 2022-07-06

**Authors:** Kai Moritz Eder, Anne Marzi, Ane Marit Wågbø, Jolanda P. Vermeulen, Liset J. J. de la Fonteyne-Blankestijn, Matthias Rösslein, Rainer Ossig, Geir Klinkenberg, Rob J. Vandebriel, Jürgen Schnekenburger

**Affiliations:** 1 Biomedical Technology Center (BMTZ) of the Medical Faculty, University of Muenster, 48149 Münster, Germany; 2 SINTEF Materials and Chemistry (SINTEF), 7034 Trondheim, Norway; 3 National Institute for Public Health and the Environment (RIVM), 3720 BA Bilthoven, the Netherlands; 4 Swiss Federal Laboratories for Materials Science and Technology (EMPA), CH-9014 St. Gallen, Switzerland

**Keywords:** Cytotoxicity, Organic nanoparticles, WST-8 cell viability assay, LDH release cell death assay, Interlaboratory comparison, In vitro

## Abstract

Nanotechnologies such as nanoparticles are established components of new medical devices and pharmaceuticals. The use and distribution of these materials increases the requirement for standardized evaluation of possible adverse effects, starting with a general cytotoxicity screening. The Horizon 2020 project “Regulatory Science Framework for Nano(bio)material-based Medical Products and Devices (REFINE)” identified in vitro cytotoxicity quantification as a central task and first step for risk assessment and development for medical nanocarriers. We have performed an interlaboratory comparison on a cell-assay matrix including a kinetic lactate dehydrogenase (LDH) release cell death and WST-8 cell viability assay adapted for testing organic nanocarriers in four well-characterized cell lines of different organ origins. Identical experiments were performed by three laboratories, namely the Biomedical Technology Center (BMTZ) of the University of Münster, SINTEF Materials and Chemistry (SINTEF), and the National Institute for Public Health and the Environment (RIVM) of the Netherlands according to new standard operating procedures (SOPs). The experiments confirmed that LipImage™ 815 lipidots^®^ are non-cytotoxic up to a concentration of 128 µg/mL and poly(alkyl cyanoacrylate) (PACA) nanoparticles for drug delivery of cytostatic agents caused dose-dependent cytotoxic effects on the cell lines starting from 8 µg/mL. PACA nanoparticles loaded with the active pharmaceutical ingredient (API) cabazitaxel showed a less pronounced dose-dependent effect with the lowest concentration of 2 µg/mL causing cytotoxic effects. The mean within laboratory standard deviation was 4.9% for the WST-8 cell viability assay and 4.0% for the LDH release cell death assay, while the between laboratory standard deviation was 7.3% and 7.8% for the two assays, respectively. Here, we demonstrated the suitability and reproducibility of a cytotoxicity matrix consisting of two endpoints performed with four cell lines across three partner laboratories. The experimental procedures described here can facilitate a robust cytotoxicity screening for the development of organic nanomaterials used in medicine.

**Graphical abstract:**

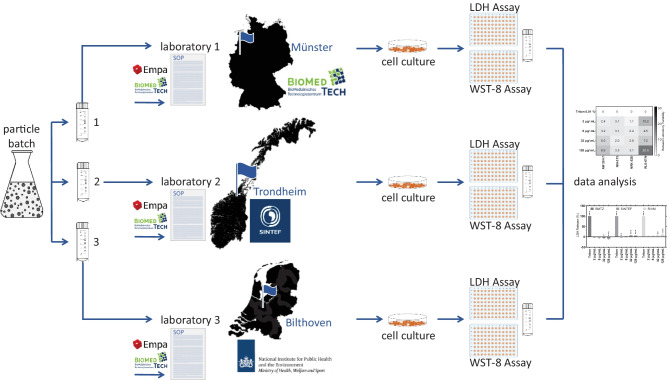

**Supplementary Information:**

The online version contains supplementary material available at 10.1007/s13346-022-01203-9.

## Introduction

Nanotechnology is a prominent topic in current research for medical devices and pharmaceuticals. The physicochemical properties and advantages of nanoparticles are utilized for enhanced drug delivery and bioimaging, as well as in regenerative medicine [1, 2]. Safety and efficacy of these nanocarriers is first determined in preclinical research on in vitro toxicity cell culture studies. Assessment of nanoparticle cytotoxicity is commonly performed by cell-based in vitro assays to evaluate their biocompatibility and safety. Reproducibility and robustness of experimental assay results is a central quality criterion for in vitro cytotoxicity assessment in the field of medical nanotechnology [3–6]. To address these issues of reproducibility and robustness and increase standardization, interlaboratory comparison methodologies can be applied to evaluate the variability of experimental results when performed in different locations by trained personnel according to standard operating procedures (SOPs). These collaborative method validation studies can be used to identify the suitability of a selected method protocol to accurately measure a certain endpoint. In the frame of the Horizon 2020 project “Regulatory Science Framework for Nano(bio)material-based Medical Products and Devices (REFINE),” interlaboratory experiments were performed by three individual partners on an in vitro cytotoxicity matrix consisting of two separate cytotoxicity assays and four cell lines of different organ origins. Organic nano(bio)materials (NBMs) are gaining importance in the field of drug delivery as nanocarriers for drug targeting [7, 8]. Three representative organic NBMs were selected as test materials for the presented interlaboratory comparison experiment, lipid-based LipImage™ 815 lipidots^®^ [9], and two variants of polymeric poly(alkyl cyanoacrylate) (PACA) nanoparticles, namely empty and cabazitaxel-loaded poly(ethylbutyl cyanoacrylate) (PEBCA) nanoparticles [10–12].

Robust, predictive, and reliable methods for cytotoxicity evaluation of nanoparticles in vitro are highly important for regulatory affairs and medical development. Also, mechanistic experiments for particle uptake and intracellular effects require standardized in vitro assays [12, 13]. The Organization for Economic Cooperation and Development (OECD) provides guidelines for in vitro cytotoxicity testing, but guidelines adapted for nanomaterial hazard and safety evaluation require further standardization and development [14, 15]. The European Commission’s Joint Research Centre (JRC) is also putting effort into standardization, development, and harmonization of assay procedures for nanomaterial in vitro cytotoxicity testing [16]. Furthermore, International Organization for Standardization (ISO) document ISO/TR 10,993–22:2017 “Biological evaluation of medical devices—Part 22: Guidance on nanomaterials” provides guiding on nanomaterial testing in medical devices for the evaluation of biological effects. An 3-(4,5-dimethylthiazol-2-yl)-5-(3-carboxymethoxyphenyl)-2-(4-sulfophenyl)-2H-tetrazolium (MTS) assay is described for in vitro measuring of nanoparticles cytotoxicity in ISO 19007, but with a focus on non-organic engineered nanoparticles. However, these efforts did not result in widely accepted and applicable standard protocols for nanomaterial in vitro cytotoxicity testing.

Several interlaboratory comparison studies have been published for nanomaterial in vitro testing [3, 17, 18]. A study from 2013 identified an approach of multiple assay endpoints and cell types of relevant and different organ origins as critical for valid results in engineered nanomaterial testing [4, 19]. The importance of interlaboratory comparison experiments was highlighted in a study performed with HepG2 cells and a number of cytotoxicity and immunological in vitro endpoints [3]. Factors contributing to successful interlaboratory comparison experiments are distribution and use of identical cell lines and sera, and well-characterized nanoparticles by all contributing partners. An interlaboratory comparison study on the widely used MTS assay for cell viability characterization reported robust assay results and low variabilities between 15 laboratories [5]. Albeit interlaboratory comparison studies on biological effects of inorganic nanoparticles have been published, similar comparative studies to test robustness of assay protocols for organic NBMs are still required [20].

A regulatory need for additional assay procedures for robust organic nanomaterial testing has been identified and communicated in the scientific community [21–23]. This lack of interlaboratory comparison for validated assays is a scientific and regulatory concern addressed in this study. Standardized and harmonized assay procedures may also facilitate development of organic nanomaterials for medical use.

In the current method pool for evaluation of NBM cytotoxicity, a number of assays are available [24]. Commonly applied are tetrazolium dye-based assays for measurement of metabolic activity of cell cultures [25–27]. The straightforward assay procedures and availability of these assays make them desirable for basic cytotoxicity assessment of nanomaterials. A combination of two or more different assays and several cell lines as experimental matrix was described to be suitable for robust cytotoxicity assessment for a larger number of engineered nanomaterials [28]. In the current study, a tetrazolium-based WST-8 cell viability assay is performed as part of the interlaboratory comparison with four cell lines. NBMs may not only affect cell metabolic activity, but also interact with cell membranes and may cause necrosis. A lactate dehydrogenase (LDH) release cell death assay is included as cytotoxicity endpoint of the assay matrix tested here [25, 28, 29]. Measurement of LDH enzyme activity released to the cell culture supernatant allows evaluation of cell death and membrane damage.

Hazard and safety characterization of nanoparticles may be limited in precision by interactions of the test agent with assay components that cause interferences [30, 31]. For this reason, multiple cytotoxicity endpoints quantified in different assays lead to more robust evaluation of toxicological potential [13, 32]. Herein, we describe an in vitro cytotoxicity testing matrix for organic nanoparticles consisting of two endpoints and four cell lines of different organ origins. Standard operating procedures (SOPs) for the WST-8 cell viability assay and LDH release cell death assay including detailed description of cell culture workflow, assay procedures, and data evaluation have been created and distributed to the interlaboratory comparison partners. Organic nanoparticles were characterized by the sponsors and identical batches of material were used for cell-based experiments in the same time frame by all laboratories, which is an important factor for data robustness. For both assay endpoints, mean percentages of the corresponding control and standard deviation of the mean are shown as indication of organic nanoparticle effect on the different cell lines. The variability of assay results is presented as standard deviation of mean within a laboratory and between interlaboratory comparison partners. This approach allows for the comparison of variability in results at the individual facilities and between the partner laboratories. In summary, we have established and validated standardized protocols for an NBM test matrix with commercially available cell lines and cell viability and cell death assays adapted for the test of organic NBMs. The described test matrix can be used in many laboratories to evaluate nanomaterial toxicity with a robust performance.

## Material and methods

Organic nanoparticle synthesis and characterization was performed by SINTEF (Trondheim, Norway) and CEA (Grenoble, France). Cell culture handling and the interlaboratory comparison assay experiments were performed according to established and within the REFINE project under the guidance of EMPA (St. Gallen, Switzerland) further developed SOPs at the three partner laboratories BMTZ (Münster, Germany), SINTEF, and RIVM (Bilthoven, Netherlands). The SOPs for the WST-8 cell viability assay and LDH release cell death assay generated within this study are available at BMTZ (Münster, Germany) upon request.

### Organic nanoparticle synthesis and characterization

In the frame of this interlaboratory comparison experiment, two types of nanomedicines were selected for testing: LipImage™ 815 lipidots^®^ [9], and polymeric nanocarriers composed of the poly(alkyl cyanoacrylate) polymer, poly(2-ethylbutyl cyanoacrylate; PEBCA). PEBCA were tested both empty and loaded with the API cabazitaxel [10]. These materials were not selected for their therapeutic or pharmaceutical use, but as a representative group of materials in current medical research. Characterization of the organic nanoparticles was performed as described in the published literature on nanoparticles synthesis.

Batches of LipImage™ 815 lipidots^®^ were prepared by high-pressure homogenization as described before [9, 33]. The lipid synthesis phase was comprised of soybean oil, Suppocire™, soy lecithin, and the fluorescent dye IR-870 oleyl [34]. LipImage™ 815 lipidots^®^ were 54.7 nm in size with a polydispersity index (PDI) of 0.10 and a ζ-potential of − 1.5 ± 1 mV. PACA nanoparticles were synthesized at SINTEF by mini-emulsion polymerization as described before [11, 12]. In short, an aqueous phase consisting of 0.1 M HCl containing the two PEG stabilizers was added to an oil phase. The water and oil phases were mixed and immediately sonicated for 3 min on ice (6 × 30 s intervals, 60% amplitude, Branson Ultrasonics digital sonifier). The pH was then adjusted to 5.0 to allow further polymerization for 5 h at room temperature. Empty PACA nanoparticles were measured to be 136.2 nm in size with a PDI of 0.11 and a ζ-potential of − 4.8 mV. Cabazitaxel-loaded PACA nanoparticles were 121.8 nm with a PDI of 0.14 and a ζ-potential of − 5.5 mV.

### Cell culture

Experiments were performed with RAW 264.7 mouse macrophages, NIH-3T3 mouse fibroblasts, NRK-52E rat epithelial kidney cells, and RLE-6TN rat lung epithelial cells. The selection of commercially available standard cell lines considers features and cellular diversity of different organs of origin and allows identification of cell type-dependent cytotoxicity [35]. Nanomaterials were shown to have different biological responses dependent on the selected cell line or the assay system [36]; thus, this matrix of four cell lines allows a more sensitive cytotoxicity analysis. As a result of our earlier research on nanomaterial cytotoxicity and cell line sensitivity, the four cell lines cultivated and tested in this study has proven to be the most sensitive and applicable for this research field [28]. The biosafety level classification of cell lines has to be regarded according to individual national law. For this reason, RIVM excluded RLE-6TN rat lung epithelial cells from their experiments. Cell lines were cultured according to the standard cell culture procedures without antibiotics [37]. Mycoplasma contamination was frequently controlled by a commercial qPCR kit (Sartorius, Göttingen, Germany). RAW 264.7 mouse macrophages (ATCC^®^ TIB 71TM, American Type Culture Collection Manassas, Virginia, USA) were cultured in Dulbecco’s Modified Eagle Medium (DMEM, Sigma-Aldrich, St. Louis, Missouri, USA) supplemented with 10% fetal calf serum (FCS, PAN Biotech, Aidenbach, Germany/Gibco, Waltham, Massachusetts, USA), 1 mM Pyruvate (Biochrom, Berlin, Germany), and 2 mM Glutamine (Merck, Darmstadt, Germany), passaged twice a week. NIH-3T3 mouse embryonic fibroblasts (ATCC^®^ CRL 1658TM) were cultured in DMEM supplemented with 10% FCS, 1 mM Pyruvate, and 2 mM Glutamine, passaged three times a week. NRK-52E rat kidney epithelial cells (ATCC^®^ CRL 1571TM) were cultured in DMEM supplemented with 10% FCS, 1 mM Pyruvate, and 2 mM Glutamine, passaged twice a week. RLE-6TN rat lung epithelial cells (ATCC^®^ CRL 2300TM) were cultured in Roswell Park Memorial Institute (RPMI, Sigma-Aldrich, St. Louis, Missouri, USA) medium supplemented with 10% FCS, 1 mM Pyruvate, and 2 mM Glutamine, passaged twice a week. For all cell lines, passages 5–20 were used for LDH release cell death assay and WST-8 cell viability assays.

### WST-8 cell viability assay

Adherent cells of the assay matrix were used (RAW 264.7, NIH-3T3, NRK-52E, RLE-6TN) that were previously cultured for 3–4 days and have grown to about 70% confluency. To harvest cells, they were washed once with PBS then incubated with trypsin solution (Gibco, Waltham, Massachusetts, USA) for 10 min (depending on the cell line and how strong adhesion is) at 37 °C and 5% CO_2_. The detached cells were transferred to a 15-mL centrifuge tube and pelleted 4 min at 37 °C and 1300 rpm (330 × g). The supernatant was removed and the cells were resuspended in cell culture medium. Cells were counted using an automated cell counter or a Neubauer counting chamber. The cells were diluted to 50,000 cells/mL for NIH-3T3, NRK-52E, and RLE-6TN and 150,000 for RAW 264.7 for seeding in a 96-well plate. One hundred microliters of the diluted cell suspension per well was applied. One column of wells was loaded with 100 μL culture medium without cells. The culture plates were incubated for 24 h in a cell culture incubator at 37 °C and 5% CO_2_. Before usage, control cell growth in the assay plate wells should exhibit 70% confluency.

After 24 h, the cell culture medium was removed from the cells, and 100 μL per well of the controls and nanoparticle dispersions was loaded to each well (all dispersions in media and control media are supplemented with 25 mM HEPES to stabilize pH). Triton X-100 was applied as cytotoxicity control with a concentration of 0.01%, complete cell culture medium as viability control, and medium without cells as absorption background. For statistical certainty, parallel wells were performed in triplicates or higher and every experiment was repeated at least 3 times. Mass concentrations of NBMs were set to 2, 8, 32, and 128 µg/mL by all interlaboratory comparison partners.

The supernatant was removed from the plates. One hundred-microliter culture medium was added to each well and removed again after 4-min equilibration. One hundred-microliter WST-8 working medium (complete cell culture medium, 0.7 mM WST-8, 1-m PMS 0.04 mM) was added to all wells. The cells were incubated for 60 min in a cell culture incubator at 37 °C and 5% CO_2_. (The optimal duration of incubation time depends on the specific cell line used and should be determined in a pre-experiment, if not yet defined. During the incubation, the positive control wells (cells in medium without supplemented nanoparticles) should reach an OD-450 nm readout of about 0.7 to 1.3 for optimal signal strength.) Before assay readout, to ensure homogeneous distribution of the colored product, the 96-well plate was mixed gently on an orbital shaker for 30 s at 500 rpm, and immediately centrifuged at 1300 × g for 1 min to remove bubbles in all wells. The concentration of reduced WST-8 in the cell supernatant increases with the metabolic cell activity and was detected based on measurements of the absorbance of WST-8 formazan. The light absorption of the reduced WST-8 was detected with a spectrophotometer at 450 nm. Measurement at the wavelength of 620 nm was used to correct in case of enhanced turbidity caused by cells and remaining nanomaterials.

The mean values and standard deviations were calculated from each series of repeated parallel reactions. Low values of OD_450nm_ may occur from culture medium and WST-8 spontaneous reactivity, which is dependent on pH, time of incubation, and exposure to light. To correct for this, the mean value of the background controls (wells without cells = medium and WST-8 background absorbance) was subtracted from all other mean reaction values. The resulting value of the medium control (cells in medium without NP = regular cell viability) was set to 100%. All other conditions were evaluated relative to the 100%—value of the negative medium control, the relative metabolic cell activity, and the associated standard deviations were calculated. Zero percent cell metabolic activity /proliferation (or 100% inhibition of metabolic activity) is represented by the background control wells (Eq. 1).

1 $$Viability\;\%=\frac{{OD}_{450-620}\;sample-{OD}_{450-620}\;(background)}{{OD}_{450-620}\;medium-{OD}_{450-620}\;(background)}\times100\;\%$$

### LDH release cell death assay

Adherent cells of the assay matrix (RAW 264.7, NIH-3T3, NRK-52E, RLE-6TN) that were previously cultured for 3–4 days were grown to about 90% confluency. Cells were washed once with PBS, then incubated with trypsin solution for 10 min at 37 °C and 5% CO_2_. The detached cells were transferred to a 15-mL centrifuge tube and pelleted 4 min at 37 °C and 1300 rpm (330 × g). The supernatant was removed and the cells were resuspended in cell culture medium. Cells were counted using a Neubauer counting chamber or an automated cell counter. The cells were diluted to 300,000 cells/mL for NIH-3T3, NRK-52E, and RLE-6TN and 600,000 for RAW 264.7 for seeding in a 96-well plate. One hundred microliters of the diluted cell suspension per well was applied. One column of wells was loaded with 100 μL culture medium without cells. The culture plates were incubated for 24 h in a cell culture incubator at 37 °C and 5% CO_2_. Before usage, cell growth in the assay plate wells was controlled to confirm confluency.

After 24 h, the cell culture medium was removed from the cells, and 100 μL per well of the controls and nanoparticle dispersions was loaded to each well (all dispersions in media and control media were supplemented with 25 mM HEPES to stabilize pH). Organic nanoparticles were incubated as described for the WST-8 assay.

After 24-h incubation, the cell culture plate was centrifuged at 270 × g for 10 min. Supernatant was carefully removed and transferred to an uncoated 96-well plate with a transparent flat bottom. The plate was centrifuged at 1300 × g for 1–2 min to remove bubbles in all wells. One hundred-microliter INT-working solution (lactic acid 56 mM, PMS 0.28 mM, INT 0.66 mM, NAD 1.3 mM) was added to each well. Subsequently, the assay plate was placed in the spectrophotometer and measurements were started (light absorption 492 nm) immediately.

Measurements were performed continuously in all wells every minute for 30 min to obtain kinetic data of LDH activity at 28 °C. LDH release increases proportionally to cell lysis; the relative activity of LDH in culture medium is based on measurements of the absorbance of reduced formazan at 492 nm.

From the measurement points of the LDH reaction, a graph was plotted and a regression line fitted into the data points. At the start of the reaction, the rate of conversion of the substrate will be proportional to the enzyme activity. For all samples, it must be ensured that the linear regression slope is only applied to the area of data points that displays linear behavior. To determinate relative toxicity, first, for all single reactions (single wells), the slope of the reaction (min^−1^) was determined as described. Mean values and standard deviation of the slopes were calculated from well repeats. The resulting value of the Triton X-100 positive control was rated as 100% LDH release and is applied as a 100% scale basis for all other values and standard deviations of the different reactions.

The slope (m) of OD_492_ of the test sample was related to the Triton X-100 control to calculate the relative LDH activity as presented in the equation below.

2 $$LDH\;leakage\;\%=\frac{m_{\mathrm{OD}492}\;sample-m_{\mathrm{OD}492}\;medium\;control}{m_{\mathrm{OD}492}\;TritonX100\;control-m_{\mathrm{OD}492}\;medium\;control}\times100\%$$

### Statistical analysis

The data presented here were produced in three interlaboratory comparison partner laboratories (BMTZ, SINTEF, and RIVM) and determined in 3 independent experiments (*n* = 3, if not stated otherwise). Statistical analysis was performed using GraphPad Prism version 8.3.0 and was oriented to ISO standards 5725–1 and 5725–2. The mean values and standard deviations of mean determined within each laboratory in the LDH release cell death assay and WST-8 cell viability assay were calculated and presented. Significances for the WST-8 cell viability and LDH release cell death assay in comparison to the corresponding control were calculated using multi-factorial analysis of variance: *p* < 0.05 (*), *p* < 0.01 (**), *p* < 0.005 (***). To evaluate the quality of the SOPs and reproducibility of experiments, both the standard deviation of the WST-8 cell viability and LDH release values within the laboratories (within laboratory variability) and the standard deviation of the values between the laboratories (between laboratory variability) were calculated. Within and between laboratory standard deviations as measure for variability of results are presented in heatmaps.

## Results

In this interlaboratory comparison pilot study for cytotoxicity assessment of organic nanoparticles, three partner laboratories performed the above described cytotoxicity quantification matrix of assays and cell lines. The assay matrix, consisting of two cytotoxicity endpoints and four cell lines, has been described before in studies on the cytotoxic potential of engineered nanomaterials [38]. The rationale behind the choice of combining two cytotoxicity endpoints and different cell seeding numbers therein is to generate a more differentiate evaluation of nanomaterial cytotoxic potential. While the LDH release assay quantifies membrane damage of the organic nanocarriers on confluent cell monolayers, the WST-8 cell viability assay quantifies the effects of the nanomaterials on the metabolic activity of subconfluent proliferating cell cultures. From the two measured endpoints, it is thus to be expected that sensitivity of the WST-8 cell viability assay is higher than for the LDH release cell death assay. The SOPs generated for organic nanocarrier testing within the project for the experimental procedures and data evaluation were circulated by BMTZ to the partner laboratories. The organic nanoparticles were synthetized and characterized as described above and distributed to the participating laboratories. Execution of SOPs was established in the participating laboratories. Mass concentration ranges of the organic nanoparticles were selected by the project consortium members with the advice of the manufacturers. The “Results” section presents first the effects of organic nanoparticles on the four cell lines quantified in a WST-8 cell viability assay, followed by the results with the same combination of nanoparticles, concentrations, and cell lines for an LDH release cell death assay. Finally, a comparison of the within and between laboratory variability in the results of the two assays is presented.

### Effects of the organic nanoparticles on cell viability quantified via WST-8 assay by the individual partner laboratories

**Fig. 1:**
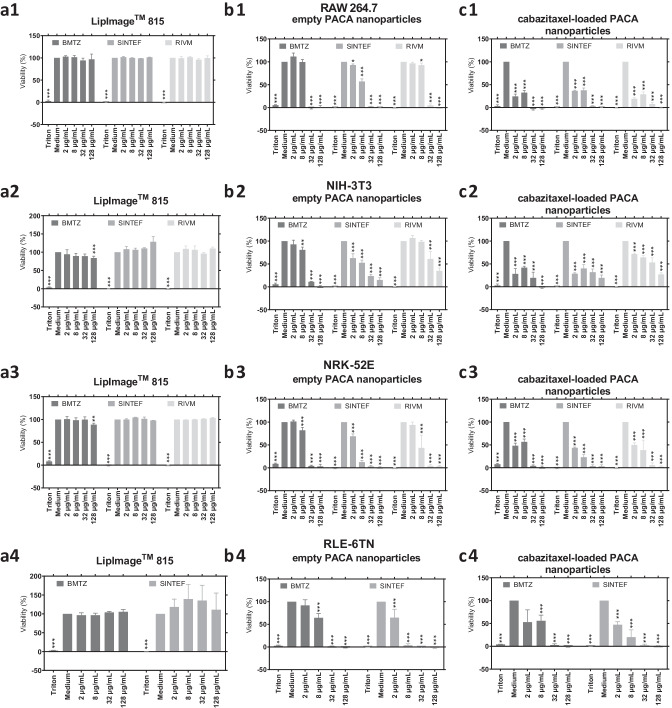
Effects of the organic nanoparticles on cell viability measured in the WST-8 assay performed by the three individual partner laboratories BMTZ, RIVM, and SINTEF. In the bar charts, mean cell viability (%) in comparison to the cell culture medium control for each combination of cell line and organic nanoparticles tested is shown. Standard deviation of mean is indicated by whiskers. Cells were incubated for 24 h with the organic nanoparticles and controls. Subsequently, a WST-8 assay was performed as described in the method section. Data was acquired from three independent experiments (*n* = 3) with up to 8 technical repeats each (*N* = 8). An ANOVA was performed for the statistical analysis of the WST-8 assay results in comparison to the cell culture medium control, and significance levels were given as *p* < 0.005 (***), *p* < 0.01 (**), and *p* < 0.05 (*)

Cell viability of RAW 264.7 macrophages, NIH-3T3 fibroblasts, NRK-52E kidney epithelial cells, and RLE-6TN lung epithelial cells upon incubation with organic nanoparticles was quantified by WST-8 assay after 24 h of incubation. Figure 1 presents the mean percentage of cell viability as determined in the WST-8 assay by the three partner laboratories for the combinations of cell lines and organic nanoparticles. In the rows (1–4) of Fig. 1, the results for the cell lines are listed, while the columns (a–c) list the organic nanoparticles tested, namely LipImage™ 815 lipidots^®^ and empty and cabazitaxel-loaded PACA nanoparticles. ANOVA was applied for statistical evaluation of significance levels in comparison to the cell culture viability control and is shown in Fig. 1 as *p* < 0.005 (***), *p* < 0.01 (**), and *p* < 0.05 (*). Supplementary Figure S1 shows the results of the WST-8 cell viability assay grouped by concentration instead of partner laboratory, with significances determined in comparison to the viabilities quantified at BMTZ.

LipImage™ 815 lipidots^®^ caused no or only slight reduction of viability in any of the four cell lines as shown in Fig. 1a1–a4. In the RAW 264.7 macrophage cell line, the mean viability varied the least of the cell lines and ranged between 94.2 ± 5.2 and 102.9 ± 2.2% when the cells were exposed to lipidots at concentrations of 2 to 128 µg/mL (Fig. 1a1). LipImage™ 815 lipidots^®^ showed the highest reduction of cell viability in NIH-3T3 fibroblasts in the BMTZ experiments with a value 84.1 ± 4.9% of medium control cells as seen in Fig. 1a2. For the NRK-52E kidney epithelial cells, viability values range from 88.7 ± 3.1% (BMTZ) at 128 µg/mL of LipImage™ 815 lipidots^®^ to 104.5 ± 0.6% (SINTEF) at 8 µg/mL. RLE-6TN lung epithelial cells showed the highest viability values up to 139.3 ± 39% across all cell lines and partner laboratories as seen in Fig. 1a4. Generally, viability values above 110% were measured for the lung epithelial cells by SINTEF. Overall, no dose-dependent cytotoxic effect of the lipid-based nanocarriers could be quantified in the WST-8 cell viability assay.

The empty PACA nanoparticles caused a reduction of cell viability below 5% at 32 and 128 µg/mL in all three partner laboratories for RAW 264.7 macrophages after 24 h of incubation (Fig. 1b1). The lower concentrations of 2 and 8 µg/mL had less impact with viability percentages from 57.1 ± 5.7 to 111.6 ± 7.6% as seen in Fig. 1b1. NIH-3T3 showed the lowest response in viability across the four cell lines with viability percentages ranging from 0.6 ± 1.8 (BMTZ) to 34.9 ± 6.4% (RIVM) at the highest concentration of 128 µg/mL. Each of the three lower concentrations of empty PACA nanoparticles caused similar effects measured in the WST-8 cell viability assay across the three partner laboratories. NRK-52E epithelial cells responded with reduced cell viability from 1.2 ± 1.4% (SINTEF) at 32 µg/mL to 4.3 ± 2.7% (RIVM) at 128 µg/mL to the two higher concentrations of empty PACA nanoparticles, while the lowest concentration of 2 µg/mL caused a maximum reduction of viability to 69.0 ± 5.2% (SINTEF). The experiments performed with RLE-6TN lung epithelial cells by BMTZ and SINTEF showed similar results as shown in Fig. 1b4 for all concentrations of empty PACA nanoparticles except for 8 µg/mL, where the viability quantified was 64.4 ± 9.6% and 3.6 ± 3.6%, respectively. Results for 32 and 128 µg/mL were in the range of 1.5 ± 0.6% and − 1.5 ± 1.7% for BMTZ and SINTEF, respectively.

Similar to the empty nanocarrier variant, the cabazitaxel-loaded PACA nanoparticles caused cytotoxicity that was quantified in the WST-8 cell viability assay by all three partner laboratories. While the two higher concentrations of 32 and 128 µg/mL caused strong effects on cell viability of more than 50%, similar to the empty variant, the effects in the two lower concentrations tested were more pronounced with the PACA nanoparticles loaded with active pharmaceutical ingredient (API). In RAW 264.7 macrophages, comparable effects were measured across the three laboratories of 37.7 ± 5.0% (SINTEF), 28.9 ± 5.2% (RIVM), and 32.6 ± 4.5% (BMTZ) at 8 µg/mL of cabazitaxel-loaded PACA nanoparticles as shown in Fig. 1c1. Of the four cell lines, NIH-3T3 fibroblasts were most resistant to the exposed concentration range of cabazitaxel-loaded PACA. Albeit, at 128 µg/mL, the viability in NIH-3T3 quantified in the WST-8 assay ranged from − 1.7 ± 1.8 (BMTZ) to 27.1 ± 3.5% (RIVM). NRK-52E kidney epithelial cells responded with reduction to less than 10% viability at the concentrations of 32 and 128 µg/mL as shown in Fig. 1c3. Mean viability values for NRK-52E at 2 µg/mL ranged from 43.6 ± 1.7 (SINTEF) to 49.8 ± 4.6% (RIVM). In Fig. 1c4, the results for the RLE-6TN lung epithelial cells incubated with cabazitaxel-loaded PACA nanoparticles performed by BMTZ and SINTEF are shown. At a concentration of 32 µg/mL and higher, the viability of lung epithelial cells is reduced to 2.9 ± 2.5% or even lower. The lowest concentration of 2 µg/mL caused a reduction of viability to 52.9 ± 27.0% and 47.3 ± 6.5% for BMTZ and SINTEF, respectively. Similarly, incubation with 8 µg/mL of cabazitaxel-loaded PACA nanoparticles resulted in values of 55.9 ± 12.3% (BMTZ) and 20.2 ± 15.9% (SINTEF).

Overall, the WST-8 cell viability assay performed by the three partner laboratories resulted in a similar evaluation of the cytotoxic potential of the three organic nanocarriers tested here. While the LipImage™ 815 lipidots^®^ caused slight effects only, both empty and cabazitaxel-loaded PACA nanoparticles reduced cell viability in all four cell lines. It is noteworthy that the cytotoxicity induced by the empty PACA nanocarriers was increased by loading with cabazitaxel as API. During this interlaboratory comparison experiment, the WST-8 assay described in the SOP was successfully adapted from BMTZ to the other partner laboratories.

### Cell death caused by the organic nanoparticles quantified by the partner laboratories in the LDH release cell death assay

**Fig. 2:**
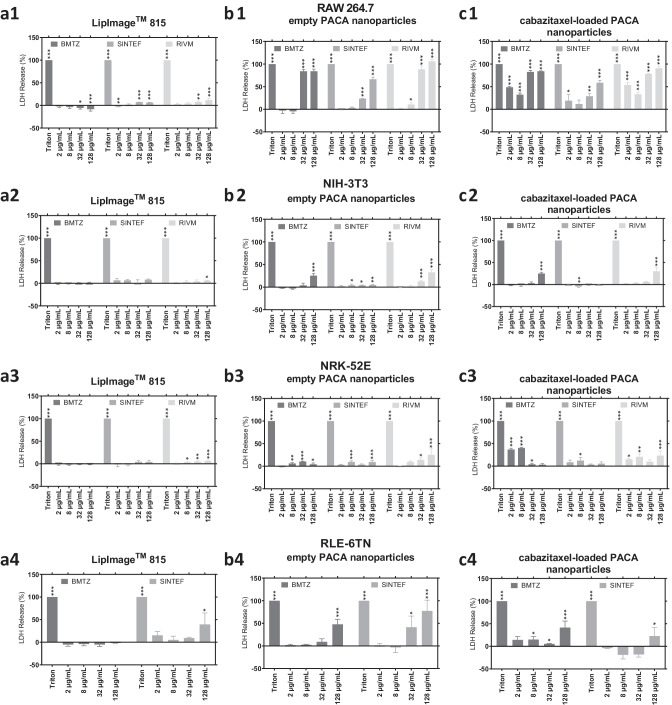
Cell death quantified via LDH release assay caused by the three organic nanoparticles in the four cell lines by the three individual partner laboratories BMTZ, RIVM, and SINTEF. In the bar charts, mean LDH release (%) in comparison to the Triton X-100 cytotoxicity control for each combination of cell line and organic nanoparticles tested is shown. Standard deviation of mean cell death is indicated with whiskers. Cells were incubated for 24 h with the organic nanoparticles and controls. Subsequently, an LDH release cell death assay was performed as described in the method section above. Data was acquired from three independent experiments (*n* = 3) with up to 8 technical repeats each (*N* = 8). An ANOVA was performed for the statistical analysis of the LDH release assay results in comparison to the cell culture medium background LDH release control (not shown), and significance levels were given as *p* < 0.005 (***), *p* < 0.01 (**), and *p* < 0.05 (*)

As described above for the results of the WST-8 cell viability assay, the three partner laboratories performed an LDH release cell death assay according to the generated SOP with the three organic nanoparticles. After cell attachment overnight, cell cultures were incubated for 24 h with four concentrations of the organic nanoparticles. Subsequently, LDH concentrations were determined in the supernatant and related to the cytotoxicity control (Triton X-100). As grouped above for the WST-8 cell viability assay, rows (1–4) of Fig. 2 include the four cell lines included in the LDH release cell death assay, while columns (a–c) list the organic nanomaterial tested. Significance levels are shown in Fig. 2 in comparison to the cell culture medium background LDH release control and is shown as *p* < 0.005 (***), *p* < 0.01 (**), and *p* < 0.05 (*). Supplementary Figure S2 shows the results of the LDH release cell death assay grouped by concentration, with significances determined in comparison to the LDH release established at BMTZ.

As presented above for the WST-8 cell viability assay, LipImage™ 815 lipidots^®^ did not induce cytotoxicity across the four cell lines tested in all three partner laboratories as seen in Fig. 2a1–a4. RAW 264.7 macrophage LDH release values ranged from − 8.6 ± 3.9 to 11.6% ± 4.1% in the concentration range of 2 to 128 µg/mL of LipImage™ 815 lipidots^®^ (Fig. 2a1). The highest LDH release values for NIH-3T3 fibroblasts were quantified at 128 µg/mL with 0.3 ± 2.1% by BMTZ, 8.1 ± 1.7% by SINTEF, and 5.9 ± 1.0% by RIVM (Fig. 2a2). Similarly, the NRK-52E kidney epithelial cells responded to the lipid-based nanocarriers with an LDH release of − 1.7 ± 1.5 to 6.0 ± 2.3% across all concentrations and partner laboratories, clearly indicating that lipidots did not affect these cell lines (up to 128 µg/mL). At BMTZ, the RLE-6TN cells also showed almost no adverse effect for all LipImage™ 815 lipidots^®^ concentrations, while SINTEF determined LDH values of 39.5 ± 25.3% at 128 µg/mL as shown in Fig. 2a4.

For empty PACA nanoparticles, we clearly measured cytotoxic effects in RAW 264.7 and RLE-6TN cell lines and all interlaboratory comparison partners as shown in Fig. 2b1 and b4. The highest LDH release values were quantified in the RAW 264.7 macrophage cell line with values of 84.2 ± 4.6%, 66.4 ± 4.6%, and 106.1 ± 4.6% at the highest concentration for BMTZ, SINTEF, and RIVM respectively (Fig. 2b1). RLE-6TN lung epithelial cells responded with 47.7 ± 11.1% and 77.7 ± 23.7% to 128 µg/mL quantified by BMTZ and SINTEF (Fig. 2b4). In contrast, NIH-3T3 fibroblasts showed no adverse response to the empty PACA nanoparticles at SINTEF, while BMTZ and RIVM established values of 25.5 ± 4.6% and 32.7 ± 5.8%, respectively, at 128 µg/mL (Fig. 2b2). RIVM determined the highest value of LDH release for NRK-52E kidney epithelial cells at 25.2 ± 13.4% at 128 µg/mL, while SINTEF and BMTZ measured values of 9.2 ± 3.8% and 4.2 ± 3.1% at 128 µg/mL and 10.2 ± 1.0% and 3.4 ± 1.8% at 32 µg/mL, respectively (Fig. 2b3).

Cabazitaxel-loaded PACA nanoparticles caused more pronounced effects at the lower concentrations of 2 and 8 µg/mL in the macrophages cell line as shown in Fig. 2c1, while the two higher concentrations showed similar LDH release values for the three partner laboratories compared to the one measured for the empty variant. NIH-3T3 fibroblasts showed no response at SINTEF, and moderate LDH release at the highest concentration at 25.1 ± 2.6% and 29.9 ± 8.4% at BMTZ and RIVM, respectively (Fig. 2c2). For the NRK-52E cells, RIVM and SINTEF measured similar LDH releases in the API-loaded PACA variant compared to the empty PACA, while BMTZ measured higher LDH releases of 36.8 ± 2.7% and 40.2 ± 1.5% for 2 and 8 µg/mL of cabazitaxel-loaded PACA nanoparticles than the other two partner laboratories. RLE-6TN lung epithelial cells were affected by the 128 µg/mL concentration in the experiments of BMTZ and SINTEF resulting in values of 41.5 ± 14.4% and 22.6 ± 19.2%, respectively (Fig. 2c4).

**Fig. 3:**
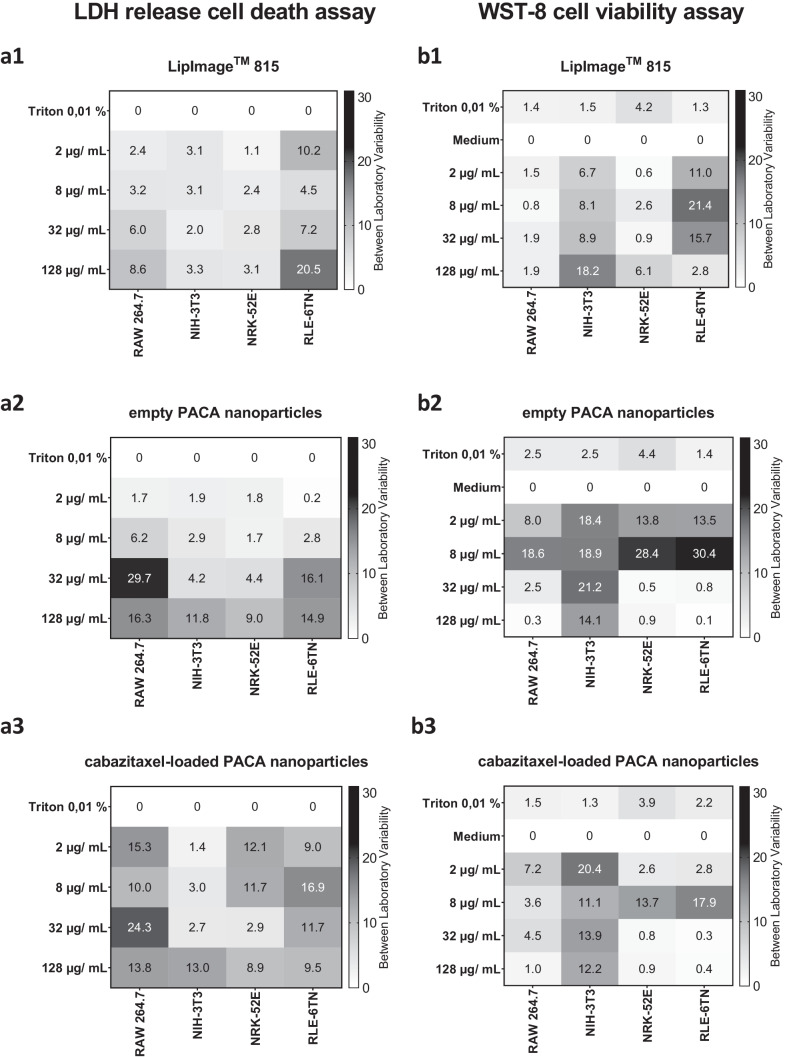
Heatmaps of the between laboratory standard deviation of results for the WST-8 cell viability assay and LDH release cell death assay performed with the four cell lines. From the data acquired by the three partner laboratories BMTZ, RIVM, and SINTEF in the two assays performed, between laboratory standard deviation was calculated and plotted. A grayscale heatmap was applied to illustrate variability of results from combinations of organic nanoparticle concentration, cell lines, and cytotoxicity assay

**Fig. 4:**
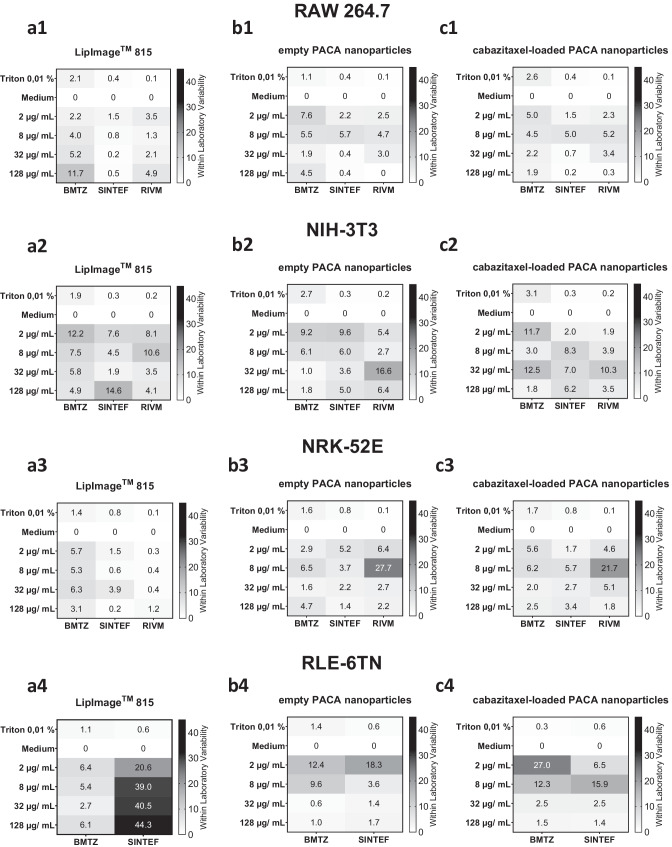
Within laboratory standard deviation of the three individual partner laboratories for three organic nanoparticles tested in the WST-8 cell viability assay. For each of the three partner laboratories BMTZ, RIVM, and SINTEF, standard deviation was plotted for the WST-8 assay. A grayscale heatmap was applied to illustrate variability of results from combinations of organic nanoparticle concentration, cell lines, and cytotoxicity assay

**Fig. 5:**
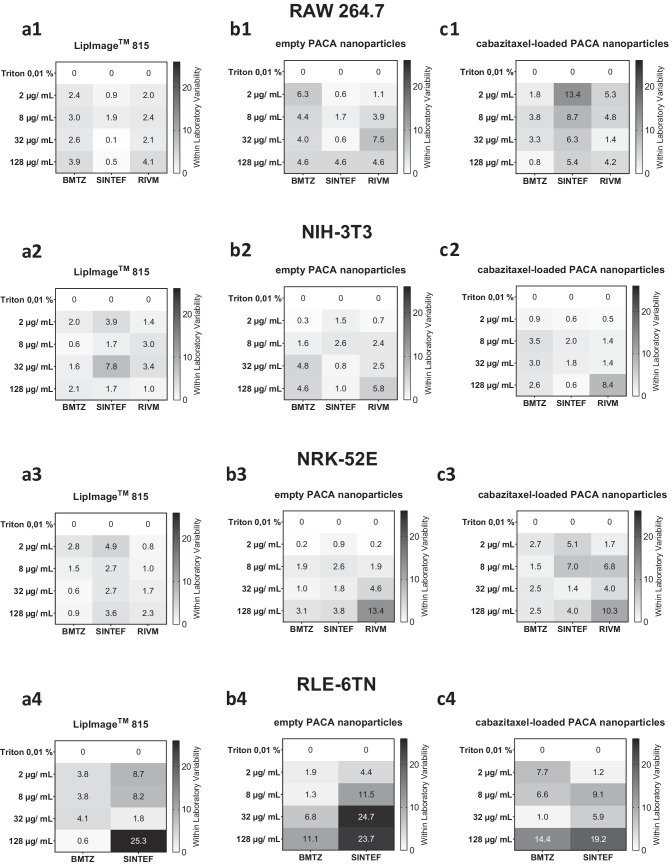
Within laboratory standard deviation of the three individual partner laboratories for three organic nanoparticles tested in the LDH release cell death assay. For each of the three partner laboratories BMTZ, RIVM, and SINTEF, standard deviation was plotted for the LDH release cell death assay. A grayscale heatmap was applied to illustrate variability of results from combinations of organic nanoparticle concentration, cell lines, and cytotoxicity assay

The LDH assay procedure and data evaluation as described in the SOP were successfully applied in the three partner laboratories to evaluate the cytotoxic potential of organic nanoparticles developed for medical use. The interlaboratory comparison of LDH release resulted in a similar evaluation of cytotoxic potential of the three organic nanoparticles. LipImage™ 815 lipidots^®^ only caused slight membrane damage in the highest concentration, while empty and API-loaded PACA nanocarriers induced cell type-dependent LDH release. Also, this quantification of organic nanoparticle cytotoxicity is in congruency with the above presented results of the WST-8 cell viability assay from the interlaboratory comparison. It is noteworthy that the cytotoxicity quantified in the WST-8 assay is generally higher because of the lower cell count in the assay in comparison to the LDH release cell death assay which is performed on nearly confluent cells. In a next step, we calculated the between laboratory and within laboratory standard deviation of all mean percentages of LDH release percentages and WST-8 viability percentages as a comparative measure for variability of results (Figs. 3, 4, and 5).

### Between laboratory variability in the cytotoxicity assay matrix

Reproducibility and robustness of the cytotoxicity assay matrix was established by determination of between and within laboratory variability. The interlaboratory comparison results for the in vitro cytotoxicity assay matrix for organic nanocarrier testing, including two assay endpoints and four cell lines, were used to calculate between laboratory and within laboratory standard deviation as a measure for variability. The standard deviations for the between and within laboratory variability are normed to the corresponding controls at each partner laboratory as the viability and cell death values. Thus, it should be noted that standard deviations are not normed to their respective endpoint and should also be considered in relation to the results of Figs. 1 and 2. The left column of Fig. 3 shows the LDH between laboratory variability data for LipImage™ 815 lipidots^®^ (a1), empty PACA nanoparticles (a2), and cabazitaxel-loaded PACA nanoparticles (a3), while the right column of Fig. 3 shows the corresponding data for the WST-8 cell viability assay in the same order (b1–b3).

The between laboratory standard deviation of LDH release values ranged from 1.1 to 20.5% and only two values are above 10% for the LipImage™ 815 lipidots^®^ as shown in Fig. 3a1. Empty and cabazitaxel-loaded PACA nanoparticles showed higher ranges of between laboratory standard deviation with 0.2 to 29.7% and 1.4 to 24.3%, respectively (Figs. 3a2 and a3). For the empty nanocarriers, five values were above 10% standard deviation, while the API-loaded variant showed 8 of 16 between laboratory standard deviations above 10%. From the illustration of the LDH result variability as heatmaps, it can be observed that no concentration dependency of cell death for LipImage™ 815 lipidots^®^ and cabazitaxel-loaded PACA nanoparticles could be shown, but empty PACA nanoparticles showed higher between laboratory standard deviation in the higher concentrations applied. Generally, the standard deviation for RLE-6TN was higher than for the other cell lines. The mean overall value of between laboratory standard deviation across the three interlaboratory comparison partners in the LDH release cell death assay was quantified at 6.3%.

Using the same approach, between laboratory standard deviation for the combinations of cell lines and organic nanoparticle concentrations were calculated for the WST-8 cell viability assay as shown in the right column of Fig. 3. Across all LipImage™ 815 lipidots^®^ values, a range of between laboratory standard deviations between 0.6 and 21.4% were measured, while four values were above 10% as shown in Fig. 3b1. Empty PACA nanoparticles showed between laboratory standard deviations from the three partner laboratories from 0.1 to 30.4% and 9 values were above 10%. Variability of WST-8 empty PACA nanoparticles results was higher in the NIH-3T3 fibroblast cell line and in the 8 µg/mL concentration in all cell lines tested (Fig. 3b2). Cabazitaxel-loaded PACA nanoparticles showed lower between laboratory variability with values of 0.4 to 20.4% and 6 values above 10% as shown in Fig. 3b3. As reported for the empty nanocarrier variant, NIH-3T3 cells showed the highest variability here. The mean overall value of between laboratory standard deviation across the three interlaboratory comparison partners in the WST-8 cell viability assay was quantified at 6.1%.

### Within laboratory standard deviation of the WST-8 cell viability assay

The above presented overall between laboratory standard deviation is now compared to variability in the individual laboratories. The within laboratory standard deviation from all experiments performed with the four cell lines is presented in Fig. 4 for the WST-8 cell viability assay. The standard deviation results are shown in the rows of Fig. 4 divided by cell lines tested (1–4) and the columns are divided by organic NBM applied to the cells (a–c). From the heatmaps, it is visible that WST-8 assay within laboratory standard deviation is cell line dependent.

RAW 264.7 macrophages showed low standard deviations across all three nanocarriers tested from 0.2 to 11.7% in the partner laboratories. The cytotoxic potential of the organic nanoparticles shows no relation to the variability of the assay results (Figs. 1, and 4a–c1). NIH-3T3 fibroblasts showed higher within laboratory standard deviation in the WST-8 assay for all three nanoparticles tested. The amount of variability in WST-8 assay results for the fibroblast cell line was not related to the particle type, cytotoxic potential, or interlaboratory comparison participating laboratory (Fig. 4a–c2). Within laboratory variability of WST-8 assay LipImage™ 815 lipidots^®^ results for the NRK-52E kidney epithelial cell line ranged from 0.2 to 6.3% for all three partners as shown in Fig. 4a3. For the empty and cabazitaxel-loaded PACA nanoparticles, within laboratory variability was similar for BMTZ and SINTEF, while RIVM showed higher variability for these combinations of cell line and nanoparticles (Fig. 4b3 and c3). Especially at 8 µg/mL of both empty and API-loaded PACA nanoparticles variants, the within laboratory standard deviation was high at RIVM with 27.7% and 21.7%. BMTZ and SINTEF showed lower within laboratory standard deviation on NRK-52E kidney epithelial cells for the empty PACA nanocarriers with values from 1.6 to 6.5% and 1.4 to 5.2%, respectively. Variability of WST-8 results was similar in the two participating laboratories for the cabazitaxel-loaded PACA nanoparticles with values from 1.7 to 6.2% over all concentrations tested. While WST-8 within laboratory standard deviation was low for RLE-6TN cells when incubated with LipImage™ 815 lipidots^®^ at BMTZ with values from 2.7 to 6.4%, within laboratory standard deviation for this combination of cell lines and nanoparticle tested resulted high variability of results at SINTEF with values from 20.5 to 44.3% (Fig. 4a4). The two PACA nanoparticles that caused cytotoxicity in RLE-6TN lung epithelial cells at a concentration of 32 and 128 µg/mL in the WST-8 assay as shown above in Fig. 1 showed low within laboratory variability at SINTEF and BMTZ at these concentrations (Fig. 4b4 and c4). The lower concentrations of empty and cabazitaxel-loaded PACA nanoparticles of 2 and 8 µg/mL had higher within laboratory standard deviation at SINTEF and BMTZ of 3.6 to 18.3% and 9.6 to 27.0%, respectively.

Overall, the RLE-6TN lung epithelial cell line showed higher within laboratory standard deviation variability compared to the other three cell lines tested. RAW 264.7 macrophages showed the most robust WST-8 cell viability assay results and good agreement in the within laboratory standard deviation across the three partner laboratories. Similarly, from the heatmaps shown in Fig. 4a–c2, it is evident that with NIH-3T3 fibroblasts comparable variabilities in WST-8 cell viability assay results are achieved by all three partner laboratories. In contrast to this finding, NRK-52E kidney epithelial cells were linked with higher within laboratory standard deviations at RIVM than at the other two participating laboratories when incubated with empty and cabazitaxel-loaded PACA nanoparticles and tested in the WST-8 cell viability assay. The mean within laboratory standard deviation in the WST-8 cell viability assay was also calculated over all nanocarriers, cell lines, and concentrations applied. This resulted in values of 5.5%, 4.1%, and 4.9% for SINTEF, RIVM, and BMTZ respectively.

### Within laboratory standard deviation of the LDH release cell death assay

The within laboratory standard deviation from all nanocarrier experiments performed with the four cell lines is presented in Fig. 5 for the LDH release cell death assay. The rows of Fig. 4 are divided by cell lines tested (1–4) and the columns are divided by organic NBMs applied to the cells by the three interlaboratory comparison partners BMTZ, SINTEF, and RIVM (a–c). In the first row of Fig. 5, the within laboratory variability of the RAW 264.7 macrophage results is shown for the three nanoparticles tested. The non-cytotoxic LipImage™ 815 lipidots^®^ were tested in the LDH release cell death assay by the three partner laboratories with very small variability. Within laboratory standard deviation ranged from 0.1 to 4.1% as shown in Fig. 5a1. In that cell line, the empty PACA nanoparticles caused slightly higher varied results in the LDH release cell death assay with values from 0.6 to 7.5% throughout the three partners, while no value was above 10% (Fig. 5b1). Similarly, the cabazitaxel-loaded PACA nanoparticles resulted in LDH assay within laboratory standard deviation of results for the RAW 264.7 macrophages from 0.8 to 13.4%, with BMTZ showing the most robust results here.

LDH release cell death assay performed with NIH-3T3 fibroblasts resulted in similar and low-level within laboratory standard deviations at SINTEF, RIVM, and BMTZ. As shown in Fig. 5a–c2, no within laboratory standard deviation exceeded 10% and heatmap distributions are similar for all three laboratories and organic nanoparticles tested. When compared to Fig. 2, it is also evident that very low LDH release percentages were measured for the NIH-3T3 fibroblasts upon incubation with the three nanoparticles for 24 h.

NRK-52 kidney epithelial cell line produced low within laboratory standard deviations when incubated with non-toxic LipImage™ 815 lipidots^®^ in the LDH release cell death assay as shown in Fig. 5a3. For this cell line, only RIVM established two within laboratory standard deviations above 10% for the empty and cabazitaxel-loaded PACA nanoparticles at 128 µg/mL (Fig. 5b3 and c3); all other values were below 10%. BMTZ quantified LDH release of 48.2% and 56.6% for the concentration of 2 and 8 µg/mL (Fig. 2c3), but within laboratory standard deviation for those measurement was low at 2.7% and 1.5%, respectively (Fig. 5c3).

RLE-6TN lung epithelial cells caused low within laboratory standard deviation in the LDH release cell death assay at BMTZ and SINTEF for LipImage™ 815 lipidots^®^ from 0.6 to 8.7%, except for 128 µg/mL at SINTEF with 25.3% (Fig. 5a4). The cytotoxic empty PACA nanoparticles caused higher LDH assay within laboratory standard deviations at the two laboratories as shown in Fig. 5b4. BMTZ showed variabilities of 1.9 to 11.1% and SINTEF from 4.4 to 24.7%. It is evident that the higher toxic concentrations of the empty nanocarrier resulted in more varied LDH release cell death assay results for the RLE-6TN cell lines. Cabazitaxel-loaded PACA nanoparticles caused within laboratory standard deviations for the LDH assay performed with RLE-6TN cell at BMTZ of 1.0 to 14.4% and SINTEF from 1.2 to 19.2%. Similar to results presented above, within and between laboratory standard deviation was the higher for the RLE-6TN lung epithelial cell line. The mean within laboratory standard deviation in the LDH release cell death assay was also calculated over all nanocarriers, cell lines, and concentrations applied. This resulted in values of 5.3%, 3.4%, and 3.2% for SINTEF, RIVM, and BMTZ respectively.

## Discussion

The nanomaterial in vitro testing strategy presented here was developed within the European project REFINE (Regulatory Science Framework for Nano(bio)material-based Medical Products and Devices) which was based on the specific tasks in the European Horizon 2020 “Call Nanotechnologies, Advanced Materials, Biotechnology and Advanced Manufacturing and Processing” to evaluate the suitability and transferability of an assay test matrix for in vitro cytotoxicity evaluation of organic nanocarriers. Also, the robustness and reproducibility of assay results and cytotoxicity measurement was determined by the comparison of within and between laboratory standard deviations for the different cell lines, assay endpoints, and organic nanoparticles. Albeit this pilot interlaboratory comparison study produced valuable data on the reproducibility and robustness of cytotoxicity assays for organic nanocarrier testing, it does not explore individual causes of variability in depth. Furthermore, a larger number of laboratories and concentrations would increase the quality of the data set. In the light of these limitations, the results presented above give an introduction on the harmonization of organic nanocarrier testing and future interlaboratory comparison experiments are needed.

Organic nanoparticles were synthetized in identical batches and distributed to the interlaboratory partner facilities to be tested in the described assay matrix [9, 11]. SOPs, generated within the frame of this study, for the experimental procedures have been circulated and implemented by all partners. Cell models, cell culture sera and chemicals, and technical equipment were used and applied as available at the partner facilities. The described approach to in vitro cell culture cytotoxicity assays with interference reducing steps and custom reagents was derived from already published and proven testing strategies for engineered nanoparticles [28]. The interference reducing experimental steps include additional washing steps for the WST-8 assay and centrifugation of cell culture supernatant for the LDH release cell death assay. In addition to these measures for controlling nanomaterial interference with the assay, which was proven suitable before [31], we have controlled the optical interference of the organic nanocarriers with both assays and the enzymatic interference with the kinetic LDH release cell death assay as shown in supplementary materials Figs. 3 and 4. It is to note that the organic nanocarriers tested in this study are designed to degrade in the neutral pH values of cell culture medium over 24 h, reducing the concern of interference in general at the 24-h endpoint [11]. Due to the similar densities of the organic nanoparticles and the complete cell culture medium with 10% serum, we conclude that the adherent cells are in steady contact during the in vitro cytotoxicity assays.

The non-cytotoxic nature of LipImage™ 815 lipidots^®^ was confirmed in the frame of this interlaboratory comparison study [9, 39]. SINTEF, RIVM, and BMTZ measured no increase in LDH release in the four cell lines when incubated with the lipid-based nanocarriers as shown in Fig. 2. Also, LipImage™ 815 lipidots^®^ only caused slight reduction of cell viability at the highest concentration in two cell lines as quantified in the WST-8 assay as presented in Fig. 1. These findings are in line with already published WST-1 data of these nanoparticles that was acquired according to ISO 10993 [9]. In the before mentioned study, LipImage™ 815 were applied to NIH-3T3 fibroblasts up to a concentration of 1500 µg/mL, while onset of cytotoxicity on fibroblasts started at 500 µg/mL. The two variants of PACA nanoparticles caused dose-dependent cytotoxicity in each of the two assays, WST-8 and LDH. Effects of the empty PACA nanocarriers quantified in the WST-8 cell viability assay were more severe in the three partner laboratories than in the LDH release cell death assay. These findings are in line with a broad study on the cytotoxicity of PACA nanomaterials performed at SINTEF and published in 2017 [11]. In that study, CellTiter-Glo^®^ assays were performed on different types of PACA nanomaterials in order to determine IC_50_ values, which ranged from 18 ± 7 (OVCAR-3 cells) to > 300 µg/mL (DU-145) for PEBCA nanoparticles, as used in this study, incubated on 12 different cell lines. Higher concentrations of empty PACA nanoparticles were necessary to trigger LDH release by membrane damage than to reduce viability of cell cultures in the WST-8 assay. RAW 264.7 macrophages responded more sensitive to the PACA nanoparticles than the other three cell lines and similar across the three partner facilities. This finding is in good agreement with published literature that nanoparticle effects are more pronounced in macrophages [35, 40]. The cabazitaxel-loaded PACA nanoparticles caused higher reduction of cell viability than their empty counterpart established by the three partners in the WST-8 assay at the lower concentrations of 2 µg/mL and 8 µg/mL. This finding is expected as a reaction to the microtubule inhibiting API cabazitaxel [41]. LDH release caused by membrane damage was quantified at all concentration for the cabazitaxel-loaded PACA nanoparticles in the macrophage cell line. The other three cell lines were less sensitive to the API-loaded nanocarriers and showed increased LDH release when exposed to 8 µg/mL or more. Cell type-dependent nanomaterial uptake may also influence the sensitivity of the different cell lines as discussed before for cytotoxic effects caused by engineered nanomaterials [28]. Macrophages are known for a rapid nanomaterial uptake in large storage vesicles [38]. Uptake mechanisms of PACA and LipImage™ 815 lipidots^®^ were investigated before on PC3 and RBE4 cells with the conclusion that both clathrin- and caveolin-mediated endocytoses occur [9, 40]. The PACA materials tested for cytotoxicity in this study were subcellularly detected by combining darkfield microscopy, confocal Raman microscopy, and ToF–SIMS analysis in NR8383 cells, confirming the uptake of these materials by macrophages [12]. A recent study on the same organic nanocarriers tested herein demonstrates the strong interaction of the particles with the immune system by a multistep in vitro hemocompatibility test [42]. The authors quantify similar trends in LDH release from donor blood as quantified in the LDH assay described above. Nanomaterial uptake of the cell lines of the assay matrix was compared by Bräutigam et al. [43]. Here, also RAW 264.7 macrophages showed a much higher degree in nanomaterial uptake than NIH 3T3 fibroblasts [43].

In summary, it is shown in Fig. 1 for the WST-8 cell viability assay and Fig. 2 for the LDH release cell death assay that the measurement of cytotoxic potential of the three organic nanoparticles is not uniform in the three interlaboratory comparison partners, but leads to similar estimation of toxicological potential. Overall, the performance on the described assay matrix is highly sensitive and robust, while effects in the cell viability assays were quantified at lower concentration than in the cell death assays. The performed interlaboratory comparison experiment was also aimed at standardization of the assay procedures and identification of important factors for reduction of variability within and between facilities.

The analysis of variability within cytotoxicity assay results revealed that within and between laboratory standard deviations are comparable and in a similar range. As summarized in detail in the right column of Fig. 3, the overall between laboratory standard deviation across the three partners and all combinations of cell lines, concentrations, and nanoparticles was 7.3% for the WST-8 cell viability assay. The individual mean within laboratory standard deviation in the WST-8 cell viability assay was calculated in the same manner and resulted in values of 5.5%, 4.1%, and 4.9% for SINTEF, RIVM, and BMTZ respectively. The mean overall within laboratory standard deviation for this assay was 4.9%. Here, it is evident that variability is slightly higher between laboratories than within each facility. This is an observation that may result from unsynchronized cellular models and cell culture sera. Cell culture sera batches and composition have been described before as a cause of variability and bad reproducibility of cell culture experiments [44, 45]. Nanomaterial quality and characteristics were closely monitored within the frame of this study, but handling of organic nanocarriers prior to cell exposures may also lead to variabilities. The experience level of laboratory personnel, e.g., technicians, PhD students, or scientists, performing the experiments is likely to affect variability of results as well. For further harmonization of assay procedures described herein, laboratory personnel shall be appointed in the SOPs. It should be noted that the SOPs used in this study do not strictly define the mode of cell counting applied in order to account for laboratory perquisites. This is another factor that could be improved for further harmonization of protocols.

For the LDH release cell death assay described herein, a similar trend of variability of results was found. Mean of between laboratory standard deviation was quantified to be 7.8%, while overall mean within laboratory standard deviation was 4.0% with individual values of 5.3%, 3.4%, and 3.2% for SINTEF, RIVM, and BMTZ respectively. Factors responsible for this observation are likely identical with the ones identified for the WST-8 cell viability assay. Here, it should be noted that the between and within standard deviations shown are normalized to their corresponding control. Applying analysis of coefficient of correlation could further illustrate the variability in relation to the measured endpoint, but would stress variability for non-toxic nanoparticles such as the LipImage™ 815 lipidots^®^. In summary, it was demonstrated that successful transfer of assay routines is indicated by similar estimates of cytotoxic potential of organic nanocarriers and between and within laboratory variability.

The fact that identical organic nanoparticles batches have been distributed and used in this study contributes to the good reproducibility of in vitro cytotoxicity assay results. Especially nanomaterial testing results can be influenced by the quality of suspensions and material variability [3, 18, 30]. Further harmonization and validation of assay procedures as described herein for organic nanomaterial testing could be achieved by implementation of standard control materials, ideally as nanosized calibration materials [46]. These nanomaterials as calibration standards would ideally have good stability and known toxicity mechanisms. For this purpose, amine-modified polystyrene nanoparticles could be applied [47]. The herein reported different sensitivities of the two cytotoxicity assays were reported before and highlight the importance of multiple test systems for accurate estimation of organic nanomaterial toxicological potential [4, 32]. A study from 2017 reported on different sensitivities of AlmarBlue, MTT, and XTT assay for testing of non-organic nanoparticles at different partner laboratories [3]. In the mentioned study, standard deviations ranged from close to 0% for non-toxic nanomaterial concentrations up to 22% for particle concentrations that caused cytotoxicity. An interlaboratory evaluation of in vitro cytotoxicity and inflammatory responses to engineered nanomaterials performed on the cell lines BEAS-2B, RLE-6TN, and THP-1 confirmed the benefits of using multiple cell lines of different species and organ origin [4]. A large comparison study on the cytotoxicity of polystyrol nanoparticles measured by the MTS assay stresses the importance of nanomaterial characterization and if possible usage of the same batches or stable commercially available standard materials [5]. The before mentioned studies also discuss that cell culture conditions are a major factor for variability of results obtained in interlaboratory comparison studies [3, 4]. This conclusion can also be drawn from the interlaboratory comparison results presented here. The macrophage cell lines RAW 264.7 produced lower within and between laboratory standard deviations in both assays even for the PACA nanoparticles that induced toxicity, while variability of results was generally higher in RLE-6TN lung epithelial cells (Figs. 3, 4, and 5). In the frame of this pilot study and as published before [35], the RAW 264.7 macrophage cell line was more sensitive to the nanomaterials than the other cell lines tested. However, previous studies using the assay matrix demonstrated that various nanomaterials caused stronger effects in other cell lines of the matrix [28]. We therefore use the matrix as a highly sensitive tool to record possible nanomaterial effects to cells. In the light of the mentioned observation, we concluded that the matrix of cell lines and assays presented herein yields additional and valuable data on organic nanocarrier cytotoxicity.

A pan-European interlaboratory comparison study from 2017 also performed cell viability assay and evaluated data as presented here in mean percentage and standard deviation of mean as percent [18]. For the MTS assay performed therein, they find between laboratory standard deviations ranging from 5 to 30% when Ag and CuO nanoparticles were tested. The authors found the MTS assay to be reproducible across laboratories, while Caspase assays and ELISA for IL1-β and TNF-α produced unacceptably high interlaboratory variability. The Joint Research Center of the EU reported IC_50_ values from 12 partner laboratories established in a colony-forming unit assay for engineered nanomaterials [16]. They report the results of variability as confidence intervals and achieve 20% intralaboratory variability and 23% interlaboratory variation over all performed experiments. The authors conclude from literature review that values below 30% are frequently considered to be an indicator for reasonable variability and affirm that the colony-forming unit assay is well transferable and reproducible between interlaboratory comparison partners. A study directed toward harmonization of an MTS assay for nanomaterial testing also reports results as EC_50_ values and states that protocol details such as cell handling, cell line ID, and media exchange are important factors to result variability [17]. The study also identified confidence intervals of 30% around the EC_50_ values across the partners as good reproducibility. The analysis of the mentioned studies suggests that testing a larger concentration range with the assay matrix described herein for organic nanocarrier testing and calculation of EC_50_ values with subsequent analysis of confidence intervals could yield additional insight on robustness of results. The authors of the studies discussed here identify cell culture sera and cell line origin as one major contribution factor for reduction of between laboratory result variability. These two factors are also underlying for the variability in the results generated herein. When compared to published interlaboratory comparison studies, the variability of results within and between partner laboratories herein is in a good percentual range for robust and reproducible assays [3–5].

The Malta initiative, a consortium of EU member states, European Chemicals Agency (ECHA), and industry partners, was founded in 2017 to support development and mutual acceptance of nano-specific testing guidelines. The above presented findings on harmonization of organic nanocarrier cytotoxicity testing form a first step toward mutually acceptable testing procedures suitable for nanomaterials. Besides this, multiple stages and barriers need to be addressed by both researchers and stakeholders of these methods [48]. The standardization and development processes will be targeted by future research projects on cytotoxicity of organic nanocarriers. Next steps toward an impact on stakeholders include an interlaboratory comparison of the assay matrix including more and complex nanomaterials and additional partners from academia and industry. Also, including a larger concentration range for calculating EC_50_ values and corresponding confidence intervals would grant additional confirmation of robustness of assay results. In the frame of this study, we demonstrated robust and reliable evaluation of organic nanocarrier cytotoxic potential in a test matrix of two in vitro assays and four cell lines. Further standardization of experimental procedures and identification of important factors for harmonized testing of organic nanocarriers were accomplished. We achieved comparable within and between laboratory standard deviations, which is an indication for successful transfer of assay routines to other facilities.

## Conclusions

Within the EU Horizon 2020 project “Regulatory Science Framework for Nano(bio)material-based Medical Products and Devices (REFINE),” the presented interlaboratory comparison study on an in vitro cytotoxicity assay matrix consisting of two endpoints and four cell lines was performed by three partner laboratories according to drafted SOPs. Cytotoxicity data and quantification of between and within laboratory standard deviation as a measurement of variability in results was produced for three organic nanocarriers. The three partner laboratories established the same dose-dependent cytotoxic potential of these particles with moderate between laboratory standard deviation. As discussed before by published literature, PACA nanoparticles were identified to cause cytotoxicity in the applied concentration range and loading with cabazitaxel increased the cytotoxic potential. Heatmaps generated for the within and between laboratory standard deviation revealed cell line-dependent patterns of variability in assay results. The low within laboratory standard deviations confirm successful implementation of assay routines according to the SOPs and robustness of the described cytotoxicity assay matrix for organic nanocarrier testing. The slightly higher between laboratory variability of results in both assays in comparison to the within laboratory variability most likely originates from unsynchronized cell lines and sera used. The robustness and repeatability of the two in vitro cytotoxicity assays is comparable within each facility and across the three interlaboratory comparison partner laboratories. In conclusion, we have established and standardized an in vitro toxicity test matrix for organic nanoparticles with commercially available cell lines and widely used cell viability and cell death assays. This easy-to-use assay matrix can be rapidly established and applied in laboratories with standard cell culture equipment and experience.

## Supplementary Information

Below is the link to the electronic supplementary material.

Supplementary file1 (DOCX 180 KB)

## Data Availability

SOPs, data, and materials may be obtained via the corresponding author upon request.
