# Supplementary material for: Standardization of an in vitro assay matrix to assess cytotoxicity of organic nanocarriers: a pilot interlaboratory comparison
Source: Drug Deliv Transl Res. 2022 Jul 6;12(9):2187–206. doi: 10.1007/s13346-022-01203-9 (PMC9360155; doi:10.1007/s13346-022-01203-9)
Supplement: Supplementary file 1 — Supplementary file1 (DOCX 180 KB) [file 13346_2022_1203_MOESM1_ESM.docx]

**Standardization of an *in vitro* Assay Matrix to assess Cytotoxicity of Organic Nanocarriers:** **A Pilot Interlaboratory Comparison**

Kai Moritz Eder^1*^, Anne Marzi^1^, Ane Marit Wågbø^2^, Jolanda P. Vermeulen^3^, Liset J.J. de la Fonteyne-Blankestijn^3^, Matthias Rösslein^4^, Rainer Ossig^1^, Geir Klinkenberg^2^, Rob J. Vandebriel^3^, Jürgen Schnekenburger^1^

^1^Biomedical Technology Center (BMTZ) of the Medical Faculty, University of Muenster

48149 Muenster, Germany

^2^SINTEF Materials and Chemistry (SINTEF), 7034 Trondheim, Norway

^3^National Institute for Public Health and the Environment (RIVM), 3720 BA Bilthoven, the Netherlands

^4^Swiss Federal Laboratories for Materials Science and Technology (EMPA), CH-9014 St. Gallen, Switzerland

*Corresponding author: kai.eder@uni-muenster.de

# Supplementary Materials

Figure S1: Effects of the organic nanoparticles on cell viability quantified using the WST-8 assay performed by the three individual partner laboratories BMTZ, RIVM, and SINTEF. In the bar charts mean cell viability (%) in comparison to the cell culture medium control for each combination of cell line and organic nanoparticles tested is shown. Standard deviation of mean is indicated by whiskers. Cells were incubated for 24 hours with the organic nanoparticles and controls. Subsequently, a WST-8 assay was performed as described in the method section. Data was acquired from three independent experiments (n = 3) with up to 8 technical repeats each (N = 8). An ANOVA was performed for the statistical analysis of the WST-8 assay results in comparison to the corresponding experiment performed at BMTZ, and significance levels were given as p < 0.005(***), p < 0.01(**), and p < 0.05(*).

Figure S2: Cell death quantified using the LDH release assay caused by the three organic nanoparticles in the four cell lines by the three individual partner laboratories BMTZ, RIVM, and SINTEF. In the bar charts mean LDH release (%) in comparison to the Triton X‑100 cytotoxicity control for each combination of cell line and organic nanoparticles tested is shown. Standard deviation of mean cell death is indicated with whiskers. Cells were incubated for 24 hours with the organic nanoparticles and controls. Subsequently, an LDH release cell death assay was performed as described in the method section above. Data was acquired from three independent experiments (n = 3) with up to 8 technical repeats each (N = 8). An ANOVA was performed for the statistical analysis of the LDH release assay results in comparison to the corresponding experiment performed at BMTZ, and significance levels were given as p < 0.005(***), p < 0.01(**), and p < 0.05(*).


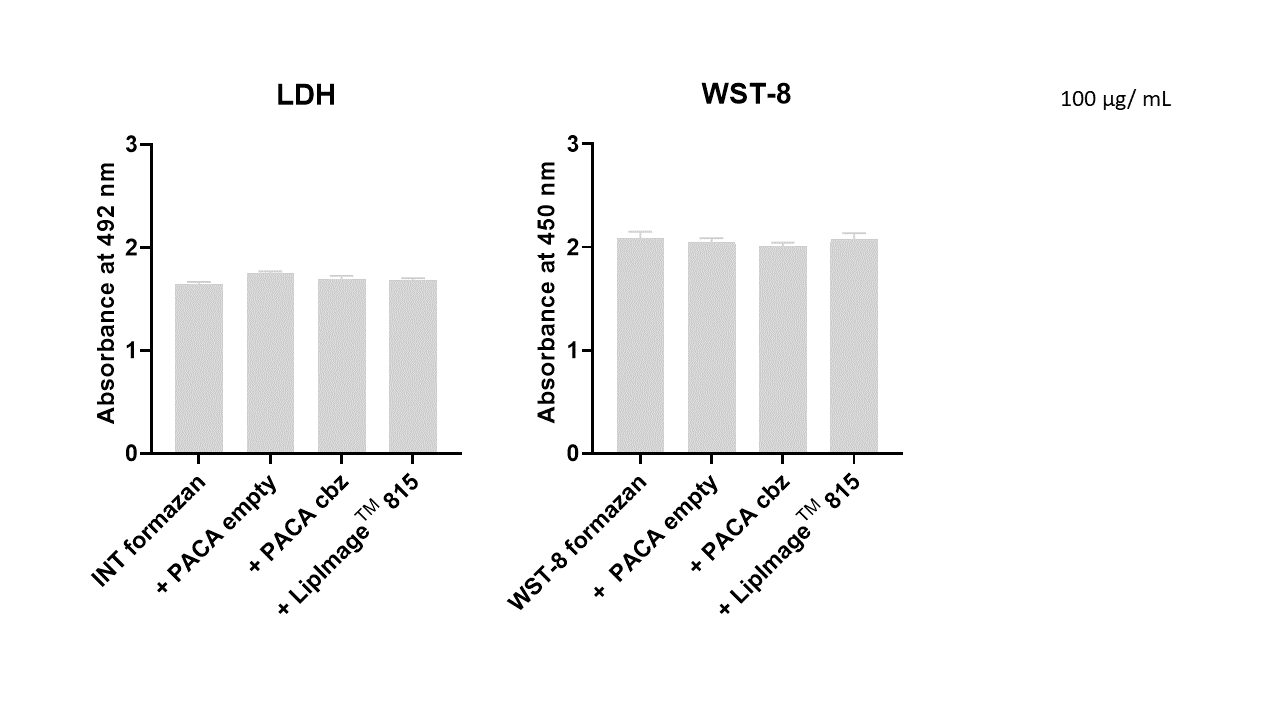


Figure S3: Optical interference of nanoparticles with WST 8 formazan and INT formazan detection. A: Absorbance of 0.07 mM WST‑8 formazan with and without organic nanocarriers. LipImage815 and empty, and cabazitaxel-loaded poly(alkyl cyanoacrylate) (PACA) nanoparticles in concentrations of 100 µg/mL. B: Absorbance of INT formazan with and without nanoparticles at 492 nm.


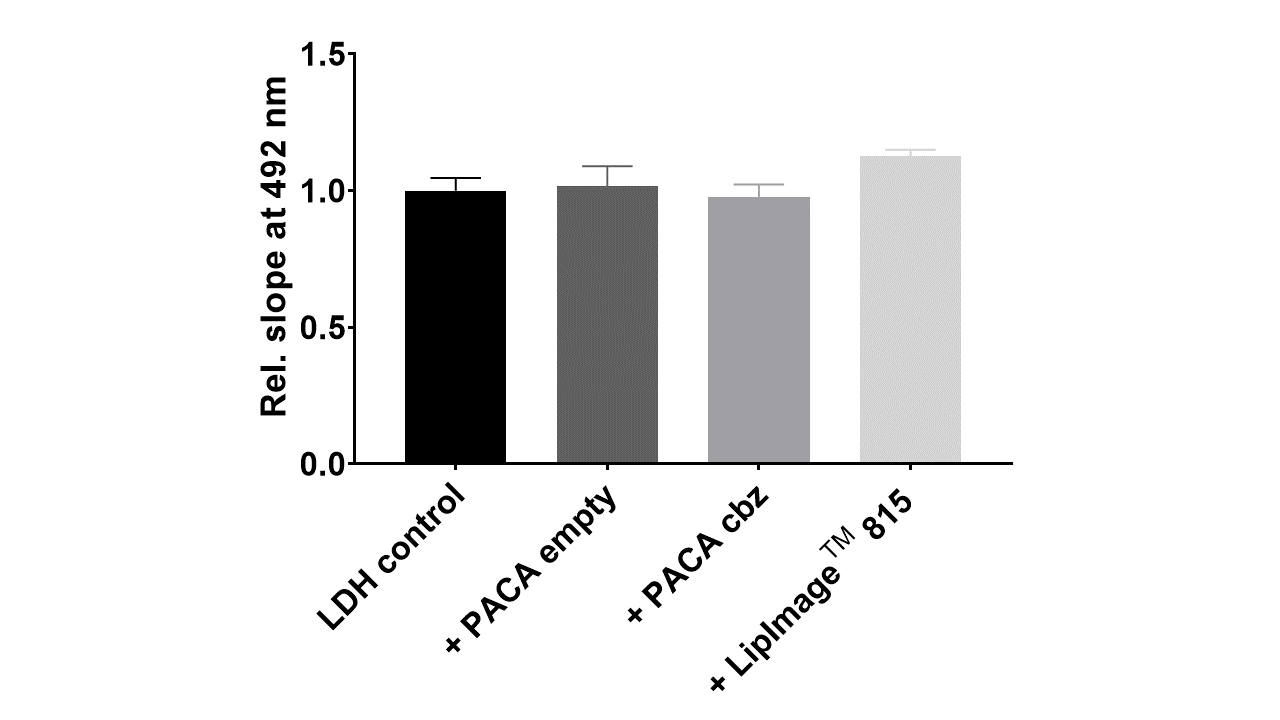


Figure S4: Enzymatic interference of organic nanocarriers at 100 µg/mL with L-Lactic Dehydrogenase. Relative slope values at 492 nm wavelength. Slope value was obtained by measurement of the absorbance every 1 minute 23 sec for 28 minutes at 492 nm. All values were related to the LDH control without nanoparticles. During the assay, the temperature was settled to 37 °C. Slope values with standard deviations were blanked on DMEM medium with INT assay solution and obtained from eight replicates each.
